# Exploring the Usefulness of a Multi-Sensory Environment on Sensory Behaviors in Children with Autism Spectrum Disorder

**DOI:** 10.3390/jcm13144162

**Published:** 2024-07-16

**Authors:** Carmela De Domenico, Marcella Di Cara, Adriana Piccolo, Carmela Settimo, Simona Leonardi, Grazia Giuffrè, Maria Cristina De Cola, Fabio Cucinotta, Emanuela Tripodi, Caterina Impallomeni, Angelo Quartarone, Francesca Cucinotta

**Affiliations:** 1 IRCCS Centro Neurolesi Bonino Pulejo, 98124 Messina, Italy; carmela.dedomenico@irccsme.it (C.D.D.); marcella.dicara@irccsme.it (M.D.C.); carmela.settimo@irccsme.it (C.S.); simona.leonardi@irccsme.it (S.L.); mariacristina.decola@irccsme.it (M.C.D.C.); fabio.cucinotta@irccsme.it (F.C.); emanuela.tripodi@irccsme.it (E.T.); caterina.impallomeni@irccsme.it (C.I.); angelo.quartarone@irccsme.it (A.Q.);; 2 Istituto Superiore G. Minutoli, 98100 Messina, Italy; grazie.giuffre@istitutosuperioreminutoli.edu.it

**Keywords:** autism spectrum disorder, multi-sensory environment, sensory integration, sensory profile, Snoezelen^®^, neurorehabilitation, rehabilitation

## Abstract

**Background**: Autism spectrum disorder (ASD) is a complex neurological development with social and communication deficits and sensory abnormalities. Sensory problems have a significant impact on daily life. Multisensory environments (MSEs), such as Snoezelen^®^ rooms, offer controlled sensory stimulation. This study aims to evaluate the effect of MSE intervention with self-controlled sensory interactions on adaptive developmental skills and sensory responses in preschool ASD children. **Methods:** This pilot study was single-blind, randomized, controlled, and adhered to the CONSORT guidelines. Twenty participants were recruited and randomized into two groups: the control group (CG) underwent treatment as usual (TAU) with individual rehabilitation sessions of psychomotor therapy. The experimental group (EG) underwent TAU integrated with the use of an MSE. Developmental abilities and severity levels were assessed, pre-post, with the Psychoeducational Profile, Third Edition (PEP-3) and the Second Edition Childhood Autism Rating Scale (CARS-2). **Results:** A significant difference in taste, smell, and tactile behaviors according to the CARS-2, as well as in gross motor skills according to the PEP-3, was observed in the EG. **Conclusions:** This pilot study suggests that MSE-integrated intervention may be a valid strategy to improve self-management of the sensory profile in autistic individuals. Further studies are needed to better identify the intervention methodology and effectiveness.

## 1. Introduction

Autism spectrum disorder (ASD) is a heterogeneous neurodevelopmental condition that is onset in early childhood. Over the last decade, an increasing prevalence of ASD has been recorded. In Asia, Europe, and North America, the average occurrence of ASD stands at approximately one percent. Specifically, epidemiological studies revealed that the average prevalence of ASD in children of 8-year-olds is around 1 in 54 in the United States, 1 in 160 in Denmark and Sweden, and 1 in 86 in Britain [1]. Similarly, in Italy, the prevalence of ASD is estimated at 1 in 77 children aged 7–9 years [2].

Moreover, the rise in diagnosed cases among females suggested that females may exhibit distinct behavioral profiles compared with males; nevertheless, ASD has remained to be diagnosed in males more frequently than females, with a ratio of 4:1 [3,4]. An earlier diagnosis may lead to earlier treatment, with benefits on development trajectories [5]; moreover, studies of early intervention suggested a substantial improvement in several developmental areas in those children who begin treatment at the preschool age [6].

According to the *Diagnostic and Statistical Manual of Mental Disorders—Fifth Edition* [7], ASD is characterized by social and communication impairment, rigid or repetitive behaviors, atypical interests, and co-occurring sensory processing problems. In this last version, sensory features were finally taken into account and atypical responses to sensory stimuli were included in the diagnostic criteria, such as the presence of hyper- or hypo-responsiveness to sensory inputs or unusual interests towards sensory aspects of the environment. Atypical sensory processing has been widely reported in ASD [8], with a described sensory reactivity symptom in 65% of autistic individuals [9]. Moreover, recent literature suggests that this abnormality extends across the individual’s lifespan, with consequent important implications in the everyday life of autistic individuals and their families [10]. This atypical processing, characterized by a difficulty in modulating, integrating, and discriminating sensory input [11], includes over- and under-reactivity to sensory stimuli and unusual sensory interests [7,12].

Hyper- or over-reactivity is defined as an excessive response to sensory input; in everyday life, it can be reflected in adverse reactions to certain types of clothing and strong reactions to touch [13,14], food selectivity [15,16], strong reactions/or avoidance behaviors to sounds [17], and lights [18]. On the contrary, common examples of hypo-reactivity may include under-responsiveness to sounds or visual stimuli [19], auditory filtering difficulties, and hypo-reactiveness to information of the environment [20]. Finally, autistic individuals may present unusual sensory interests characterized by sensation seeking, such as sniffing objects or staring intently at moving objects [21].

Specific sensory differences in individuals with ASD can often result in highly disabling distress reactions and are predictive of social dysfunctions [20]. The severity of these symptoms can have a negative impact on daily life by interfering with various common situations and hindering adaptive behaviors [22]. Several studies have described difficulties during meals [23], during school time [24], or during sleeping hours [25], and greater sensory dysfunction may be associated with an increase in challenging behaviors and worsened integration and social participation [26,27,28]. Considering the relationship between sensory processing and adaptive skills, a correct assessment and a tailored intervention seems to be necessary. Indeed, this kind of intervention is often requested by parents of children with ASD [29]. In contrast, despite the perceived need of families, insufficient evidence exists for a therapeutic approach to sensory difficulties.

In recent years, multisensory environments (MSE, also called sensory rooms or Snoezelen^®^) have been widely used, and there has been growing interest in the use of MSE for children with autism. Since its conception in 1975, the Snoezelen^®^ rooms were developed as a multisensory environment, and designed to provide multiple stimulation opportunities that cover all sensory channels [30]. His philosophy was based on non-directive and non-threatening approaches [31]. This multisensory approach originated and developed in the Netherlands, and its name comes from two Dutch words: “snuffelen”, meaning to search or explore, and “doezelen”, meaning to relax [31,32,33]. 

The usefulness of MSE was reported in different psychiatric and neurological conditions. Shapiro et al. [34] reported a reduction in the frequency of maladaptive behaviors in children with intellectual disabilities. In addition, a similar improvement was reported by Lotan on individuals with Rett syndrome [35]. Furthermore, the support of MSE was evaluated in relaxing or stimulating patients with dementia; the reported evidence underlying an improvement of engagement and beneficial effect on mood [36,37].

In the recent literature, different research groups applied similar multisensory settings to a wide range of therapeutic aims in autistic individuals. In the pediatric population, the majority of experimental interventions focused mainly on repetitive and stereotyped behaviors [38,39] and prosocial behaviors [38,40]. Based on behavioral observation, while there was a significant reduction in frequency and intensity of stereotyped behaviors, in contrast, inconsistent results were reported for spontaneous social interactions. Similar results were reported in autistic adults [31,40,41]. In the field of sensory intervention through MSE, only Mey et al. [42] showed a better adaptation to sensory stimuli in a limited population of autistic children. Moreover, Unwin [38] explored the usefulness of providing control on sensory equipment to autistic subjects; interestingly, their findings suggested that this condition may help to improve learning and engagement. Afterward, the same group showed that the multi-sensory environment equipment preferences of autistic children are related to individual differences in sensory stimuli [43].

In this pilot study, we proposed an intervention based on sensory activity in the MSE, tailored to individual children’s preferences, to support neuro-psychomotor rehabilitation. The aim was to explore the efficacy of behavioral intervention in MSE on abnormal sensory responses and adaptive behaviors in children with ASD.

## 2. Materials and Methods

### 2.1. Study Design 

The study was a single-blind, randomized, controlled pilot study. This research was carried out in accordance with the Declaration of Helsinki and gained the approval of the Ethic Committee IRCCS Sicilia Centro Neurolesi “Bonino-Pulejo”. It adheres to CONSORT guidelines and has been registered at http://www.clinicaltrials.com (accessed on 5 June 2023) (identifier: NCT05879952). Before the beginning of the research, written consent was obtained from the parents/caregivers, since the users of the daycare centers are underage subjects and are all taken care of by their parents. Following a neuropsychiatric examination, children who obtained a diagnosis of ASD satisfying the diagnostic requirements of the DSM-5 were selected and recruited. Twenty subjects participated in the study, randomized into two groups: (1) the control group (CG) underwent treatment as usual (TAU), consisting of standard neuro-psychomotor training. The treatment was tailored according to each child’s goals, needs, and preferences. Overall, each patient was treated for 4 months, a total of n = 36 sessions, twice a week, lasting 45 min each. (2) The experimental group (EG) underwent treatment as usual (TAU) integrated with the utilization of a multisensory room, in a 1:1 ratio. All exercises were tailored by therapists to meet individual treatment requirements, adjusting the level of difficulty to suit each patient’s abilities. Participants in the experimental group attended a total of 36 sessions, held twice a week over a span of 4 months. Each week, one session comprised the TAU intervention (18 sessions), while the other session was augmented with a multisensory room experience (18 sessions). Both types of sessions were 45 min in duration.

### 2.2. Inclusion/Exclusion Criteria and Participants 

The inclusion criteria for all study participants were: (a) age between 3 and 6 years of age; (b) ASD diagnosis based on the DSM-5 autism diagnostic criteria evaluated by expert psychiatrists in the field [7]; (c) signed parent/caregiver consents; (d) participants being able to attend the sessions regularly, (e) no hearing, visual, or physical disabilities that would preclude participation in the intervention, (f) absence of other major medical conditions such as epilepsy; (g) children who did not undergo any other therapeutic intervention during the study. The exclusion criteria were: (a) age not between the range of 3 and 6 years; (b) failure to fulfill diagnostic criteria of ASD; (c) lack of release of informed consent; (d) presence of other major medical conditions such as sensory deficits, epilepsy, genetic syndrome, and traumatic brain injury; (e) children who were already undergoing other therapeutic interventions during the study.

No exclusion criteria based on the level of development assessed with the PEP3 were applied, and neither minimum nor maximum thresholds were set. This approach was chosen to ensure the inclusion of a heterogeneous population, aiming for more representative and generalizable results. By including children at various developmental stages, we could better understand the intervention’s impact across a broader spectrum of abilities. Furthermore, including children at an early stage of development enables the monitoring of their progression over time. Even if they are unable to perform the task at the initial assessment, they may acquire the necessary skills during the treatment or follow-up period. This flexibility in inclusion criteria enhances the study’s capacity to capture diverse developmental trajectories and outcomes.

**Figure 1:**
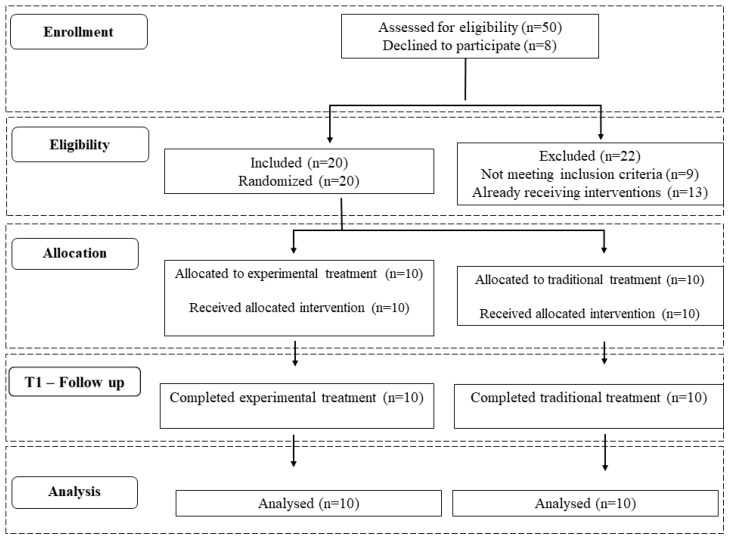
The CONSORT flow-chart with detailed information about participants in the study.

Participants were screened for eligibility at the IRCCS Sicilia Centro Neurolesi “Bonino-Pulejo”; each included patient underwent a complete medical evaluation performed by expert clinicians in the field. Successively, children who met inclusion/exclusion criteria were enrolled consecutively and randomly divided into the experimental or control group. The randomization process was supported by a computer-generated list of arbitrary numbers, allocated by a blind researcher who did not participate in the trial. For more details, see the CONSORT flowchart in Figure 1.

### 2.3. Outcome Measures

This pilot study was designed to comprehensively assess all areas of development and severity levels of core autism symptoms. Functional areas were assessed before (T0) and after (T1) the treatments by a blind multidisciplinary team composed of specialized psychologists who had attended adequate training. Primary outcome measures were: (1) Psychoeducational Profile, Third Edition (PEP-3), Italian version, a global scale to assess developmental skills and behaviors of children with autism and communication disabilities, aged between 6 months and 7 years. It identifies learning strengths and emerging abilities concerning communication, motor skills, and maladaptive behaviors [44]. In the final analysis, we used scores relating to the developmental age of the six performance subtests, to better estimate the level of children and their progress in each area. (b) Childhood Autism Rating Scale Second Edition (CARS-2), Italian version, a tool to recognize autism and establish the level of severity [45]. In our study, given the characteristics of the participants, we used the CARS-2 ST section. The CARS-2 evaluates the child’s behavior in several areas, including verbal and nonverbal communication, socialization, stereotyped and repetitive behavior, and sensory interests. It consists of 15 questions with a rating scale ranging from 1 to 4, where 1 indicates normal behavior and 4 indicates highly anomalous behavior. It is possible to obtain a global score between 15 and 60, where a higher score indicates a greater severity of ASD symptoms. The scores obtained for each item by the direct observation of children were analyzed. The pre-treatment assessment (T0) occurred just before the initiation of the 4-month treatment period, while the post-treatment assessment (T1) was conducted immediately after its completion. This timeline allows for an evaluation of the effects of the treatment immediately after its conclusion and aligns with the duration of the intervention.

### 2.4. Standard Treatment 

The standard treatment, commonly used in Italy for preschoolers, consisted of neuro-psychomotor intervention; this therapeutic option was developed by Aucouturier and Lapierre [46] and in other European countries is known as play therapy [47,48,49,50]. This approach aims to support a comprehensive intervention characterized by a developmental approach, based on the principle that it is necessary to consider each child’s play or activity preferences to encourage the development of communication, socio-relational skills, as well as cognitive, and the generalization of learning in different contexts [51]. Thanks to the variety of games and tools available in the therapeutic room, the child was stimulated through multiple sensory channels (visual, auditory, tactile, etc.). The games for children were placed on high but visible shelves, to encourage the use of gestural and visual channels, promoting the request towards the adult. The initial and final routines consisted of always the same actions and activities aimed at scanning time and activities, promoting a sense of security and tranquility. This treatment not only improves cognitive performance and supports learning, but also promotes eye contact, joint attention, shared fun, stimulating communication, and reducing restricted and repetitive behaviors [52]. Moreover, through a variety of sensory activities such as massages, sand play, tactile exploration, and balance games, the therapist gradually stimulates the child’s sensory systems in a controlled manner. This allows the child to experience and regulate their sensory reactions, ultimately improving their capacity to process and integrate sensory information from their surroundings.

### 2.5. Multisensory Room Setting 

**Figure 2:**
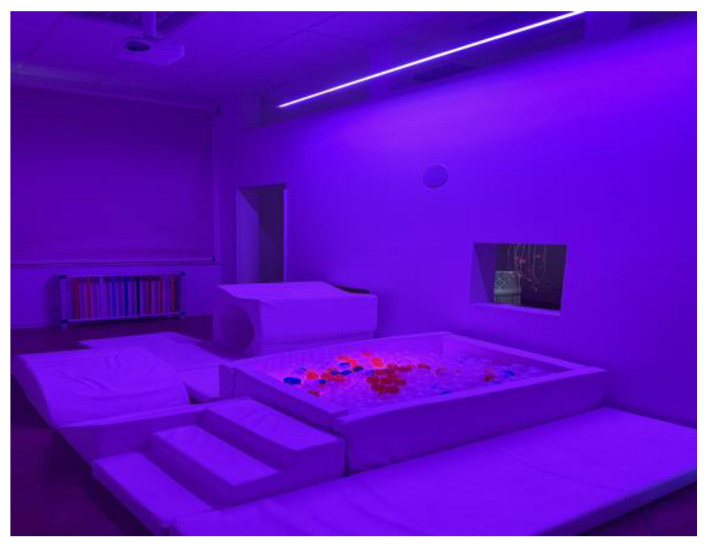
The child-size pool with luminous balls.

**Figure 3:**
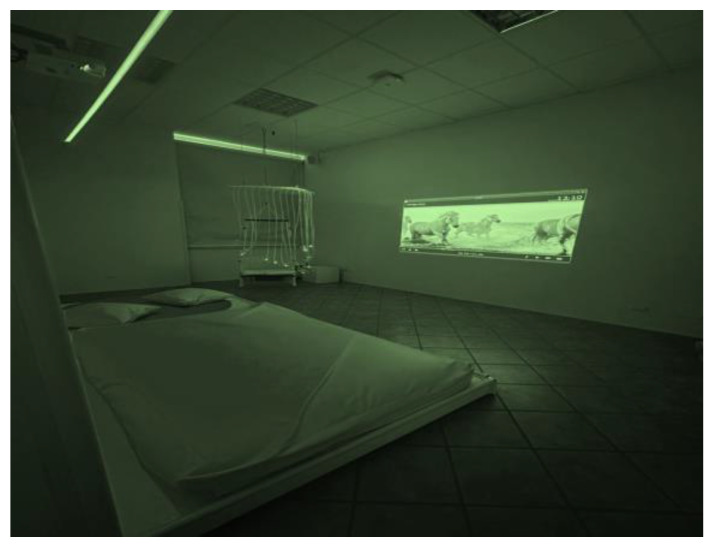
A relaxation area where a water mattress and a projector.

All the interventions were delivered by IRCCS Sicilia Centro Neurolesi “Bonino-Pulejo”. The setting used (about 30 m^2^) consists of a multisensory room designed and created by Duit S.r.l (Roma, Italy) (Design for User Innovation Technology), an Italian company located in Florence specialized in creating multisensory environments (https://www.duitfor.com/). In collaboration with Duit, we developed an innovative project for children with the aim to stimulate adventure and relaxation through the senses, with immersion in personalized scenarios. The multisensory room is composed of a unique environment integrated into two areas dedicated to interaction and relaxation, connected to each other, with the possibility to set sessions to treat only one patient at a time or simultaneously different patients. The different interaction areas allow to make up a multisensory experience where the patient can freely perform in different directions experimenting with combinations of play through: (i) a psychomotor space structured by a luminous construction composed of soft elements with different shapes and heights, a tunnel, a child-size pool with luminous balls; (ii) a relaxation area with a huge sound vibrating platform, where a water mattress and several cushions and ottomans are placed, to develop proprioception and vestibular, move, lie down and roll, playing with one’s body and with others, also favoring relationships and communication; (iii) niches to discover an experience smell and touch at different heights, also connecting the platform to the soft area. The sensory room is provided by an advanced technological system that allows the personalization and transformation of the environment, adapting it to the specific needs of each patient (a very important aspect for example in subjects with strong sensory sensitivity, such as in cases of ASDs). This system provides simplified instruments for managing and controlling the environmental context and individual sensory stimulations, integrated and regulated in a separate and totally personalized way to respectively provide the operator with an easy-to-use tool that can be flexibly adapted to different needs, and to the patient a tool to become the director of his space, choosing and adjusting light, in color and intensity, videos, music, sound vibration, even in intensity and volume. Through an external iPad, the operator can insert the most suitable videos and music based on the individual preferences of the patient or the therapeutic path studied, through a simple USB or by downloading them directly from the internet (Figure 2 and Figure 3). Additional tools expanding the range of sensory integration activities are: (i) a projector presenting images on the wall from rotating disks purposely designed for the sensory room (nature scenes—dolphins and the underwater world); (ii) a rotating color-changing disk displaying spotted light with special light effects; (iii) an aromatherapy spray emitting essential oil fragrance to stimulate olfactory receptors; (iv) a fiber optic thread 2 m long, with a density of 150 strands, safe to touch, bite, fold (when folded it makes fascinating sparkling effects); (v) an interactive bubble tube, 1.5 m tall, placed on a cushioned platform in the corner between two mirrors (110 cm × 150 cm). Further tools present in the multisensory room are a swing covered with colored optical fibers with a vibrating chair, UV lamps, auditing stimulating speakers in the room corners with sounds of nature, such as sea, ocean, heart beating, rain, water, streams, leaves, wind, bird chirping). Several of the stimuli listed above were chosen for experimental intervention based on data present in the literature, such as the use of project images and lights on the wall [40,42,53] the aromatherapy spray [53,54], and the fiber optic thread [38,42,54]. Other kinds of stimuli were also chosen, including objects or toys used in conventional clinical rehabilitation practice because highly motivating.

### 2.6. Experimental Treatment with Multisensory Room

We designed a neuro-psychomotor intervention combined with the use of a multisensory environment. Each child participated in semi-structured sessions lasting approximately 45 min. Each session began with 5 min of free exploration in the room and then continued with the possibility of carrying out 5 different activities (See Table 1) starting from the child’s attraction. The preferential choice of the children represented the rehabilitator’s first step in engaging in the activity. Furthermore, the child was allowed to control the sensory equipment himself and calibrate the intensity, duration, and frequency of the sensory stimuli, by changing the sensory aspects of the equipment using a tablet or other user-friendly devices. The operator paid attention to the duration of each activity (max 10 min) and the execution of all activities for each session. The activities carried out in the experimental group concerned the different sensory processes. The therapist created, for each tool within the multisensory environment, similar activities that engage the child’s participation and sensory processing. All activities were designed to provide a suitable challenge based on the child’s emerging skill repertoire. The rehabilitator’s intervention consisted of supporting the zone of proximal development, thus calibrating the expected levels of difficulty provided in the specific sensory domain.

### 2.7. Statistical Analysis 

Statistical analysis was performed by using version 4.3.0 of the open-source software R, considering *p* < 0.05 as the significance level. Given the small sample dimensionality, we performed a non-parametric analysis. Therefore, we used the Mann–Whitney *U* test to compare test scores between the two groups, as well as the Chi-squared test to compare proportions between the two groups. Continuous variables were expressed in the median ± first-third quartile, whereas categorical variables were in frequencies and percentages. We performed the analysis of covariance (ANCOVA) to assess whether changes in clinical outcome at T1 were influenced by treatment type (EG vs. CG), regardless of patients’ score at baseline. In detail, the dependent variable was the test score at T1, the independent variable was the categorical variable ‘Group’ (1 = EG; 0 = CG), and the test score at T0 was set as a covariate. We used ANOVA to verify whether such a model was significantly different from the one fitted with the interaction term “Group * test score at T0”. 

## 3. Results

Fifty children with ASD were screened for eligibility since September 2019. Among those, n = 9 did not meet inclusion criteria for other medical conditions and n = 13 were already receiving interventions. A total of n = 20 patients fulfilled inclusion criteria and were enrolled, 17 males and 3 females, aged 3–6 years. The age range of the sample fell within the subjects attending the Child Neuropsychiatry service of the IRCCS “Bonino Pulejo” Neurolesi Center in Messina, Italy (Table 2). 

No significant difference in age between the two groups was found (*p* = 0.85). As shown in Table 3 groups no significant differences at baseline between the two groups emerged, except for VC and IM (*p* = 0.024).

ANCOVA assumption of homogeneity of variance was not met in UO, VC, and NVC scores. The interaction term “Group * test score at T0” was significant only for gross motor skills in PEP-3 (t = −3.13; *p* < 0.01), therefore it was not considered in the remaining ANCOVA models fitting. The results of this analysis are reported in Table 4. They showed that the effect of the two treatments was significantly different in the CARS-2 TS score (t = −2.30; *p* = 0.03) and gross motor skills in PEP-3 (t = 3.09; *p* < 0.01).

## 4. Discussion

The MSE was described as such a useful setting for intervention in children with neurodevelopmental disorders [38,40,42,55,56,57]. The few trials conducted to date for ASD interventions suggested an improvement in sustained attention, improvement of developmental skills, and challenging behaviors [31,38,40,53,54]. We explored the effectiveness of a comprehensive neuro-psychomotor intervention integrated with a self-controlled over the sensory exposition of MSE in children with autism.

An integrated intervention using a multisensory room could be as effective as a standard treatment because both types of interventions aim to provide specific benefits, but through different approaches [58]. However, our study supports the idea that an MSE-based intervention yields a positive effect on the sensory behavior of autistic individuals. In contrast to previous descriptions by other authors [53], we did not find an overall improvement in the severity of autism symptoms, but only a specific and targeted improvement in behaviors inherent to gustatory, olfactory, and tactile sensory seeking. Although the common use of MSE, we attributed these dissimilarities to the strong difference in both sample and methodology between the two studies.

Specifically, we registered an improvement in sensory behavior about taste, smell, and touch. Most of the literature has focused on global improvement and challenging behaviors and few studies, to date, have explored the effect of MSE on sensory behaviors. The relevance of these findings can be interpreted by considering the importance of managing sensory sensitivities in autism [22,59]. Several studies have found that responses to unpleasant stimuli when expected, predictable, and self-selected are more likely to be perceived as pleasant; in contrast, aversion persists if these same stimuli are controlled by others [24,60]. Indeed, consistent with the findings of Unwin et al. [61], we described a significant reduction in sensory and seeking behaviors with targeted and highly personalized use of the MSE.

Several factors may have played a role in this outcome; the first reason is probably due to the difference in setting: integrating a conventional treatment with the use of a MSE could enhance therapeutic effects by offering a wider range of stimuli and opportunities for the patient with synergistic benefits. Although conventional therapy may actively involve the patient in familiar activities, MSE offers a combination of different sensory modalities, can provide a unique therapeutic experience, and can promote the exploration of new items and new skills, encouraging interaction and engagement [42,61,62]. Moreover, neuro-psychomotor interventions use specific behavioral, educational, and developmental approaches with common toys and objects, in a comfortable room without any control from children: in a conventional therapy setting, sensory stimulation is not controllable by the child; besides, the use of MSE may offer a controlled environment capable of calibrating the frequency, intensity, and duration of sensory stimuli; this can lead to a decreasing in defensive sensory behaviors due to sensory overload and may help create better conditions for learning [24,38,63,64]. Finally, a specific intervention on the patient’s sensory profile may be easier both because of the different sensory experiences of which the MSE is composed and because they can be modulated as needed, based on the singular and heterogeneous profile of each child [18].

## 5. Limitations

The main limitation of the present study is the small sample size. However, this was a pilot study and the information collected may be useful to plan future trials with a larger sample and targeted intervention. In addition, the age range of our sample could show significant differences in developmental skills between 3- and 6-year-olds, although the samples were well distributed among ages 5. Furthermore, the prevalence of male subjects was higher compared to females (M:F = 5.7:1), however, this ratio appears to be only slightly increased from the known male predominance of ASD diagnoses [2,65]. About outcome measures, we did not take into account maladaptive behaviors subscale of PEP3, because it was based not only on challenging behaviors but also considered areas such as adaptive behavior and personal autonomy, goals that were beyond the scope of our study.

The study was single-blinded, which could be considered a limitation. However, this choice should not have affected our results, since the outcome assessments were based on objective measures collected by experienced blinded assessors. Finally, there are no behavioral data collected by video recording in our study to code the child’s behaviors during the sessions. However, to our knowledge, this is the first study to use standardized and validated tests to assess change in behaviors and sensory responses through standardized tests.

Our results might suggest the differential effectiveness of the treatments, but further research with larger samples and additional controls could confirm and deepen these findings. In addition, it would be interesting to record the frequency of sensory defensive behaviors, behavioral reactions to the proposed stimuli, and the child’s communicative intentionality during the sessions. It would be interesting to also try to describe the number of changes required by the patient within the setting and how these change with exposure.

## 6. Conclusions

This pilot study suggests that MSE can be a suitable therapeutic setting; moreover, the integrated intervention using the MSE may offer opportunities for developing and learning to better manage one’s sensitivities or reactions to specific sensory stimuli. These preliminary results could pave the way for more detailed treatment considerations for the management of sensory sensitivities in autism, potentially providing important directions for care to improve children’s quality of life. However, it is essential to note that further larger-sample studies are needed to assess treatment modalities and effectiveness in specifically addressing sensory sensitivities; furthermore, the generalization and duration of these behavioral changes and the associated change in quality of life will need to be fully investigated and understood.

## Figures and Tables

**Table 1:** Description of the sensory activities used in the multisensory environment.

Description of the Sensory Tool	Type of Stimulation	Editable Element	Task
Projector	Visual–Auditory	Choice of scenario, adjustment of sound volume	Naming and classifying colors, objects, animals
Aroma spray	Olfactory	Exploration of little bottles with odors	Odor recognition, naming and discrimination
Luminous optical fiber	Tactile–Visual	Tactile manipulation and colors choice	Recognition, naming and discrimination of shapes, textures, and colors
Ball pool	Tactile–Visual	Tactile manipulation and colors of light	Numbering and color discrimination
Soap bubbles	Tactile–Visual	Visual exploration, grasping	Activation of joint play and mutual gaze.

**Table 2:** Demographic characteristics of participants.

	All	EG	TAU	*p*-Value
Participants (N)	20	10 (50.0)	10 (50.0)	-
Age (months)	51.0 (39.7–60.2)	49.0 (37.5–59.2)	51.0 (41.0–59.0)	0.849
Gender-Male	17 (85.0%)	9 (90.0%)	8 (80.0%)	0.999
CARS-2 Total Score		38.2 (35.6–45.4)	46.5 (42.0–49.7)	0.112
PEP-3—PVC		57.5 (35.2–79.7)	34.5 (24.7–53.5)	0.140

Quantitative data are in median (first-third quartiles), and qualitative data in frequencies (percentages). Legend: CG = Control Group; TAU = Subjects in treatment with TP.

**Table 3:** Statistical comparisons of clinical scores between groups at baseline.

Assessment		EG	TAU	*p*-Value
CARS-2	TOTAL	38.2 (35.6–45.4)	46.5 (42.0–49.7)	0.112
	RP	3.5 (2.6–3.5)	3.7 (3.0–4.0)	0.329
	IM	3.2 (1.6–3.5)	3.5 (3.0–4.0)	0.152
	ER	3.0 (1.6–2.6)	3.2 (2.6–4.0)	0.177
	UB	2.7 (2.1–3.0)	3.2 (2.6–3.5)	0.105
	UO	2.5 (1.7–2.5)	3.0 (2.5–3.5)	0.054
	AC	3.0 (2.6–3.4)	3.2 (3.1–3.5)	0.369
	VR	3.0 (2.1–3.0)	2.5 (1.6–2.9)	0.372
	AR	2.7 (2.1–3.0)	2.0 (1.6–2.5)	0.279
	TS	2.5 (2.0–2.5)	2.2 (2.0–4.0)	0.843
	FA	2.2 (2.0–2.5)	3.0 (2.0–3.5)	0.388
	VC	3.0 (2.6–3.4)	4.0 (3.5–4.0)	**0.024**
	NVC	2.7 (2.1–3.0)	3.7 (3.0–4.0)	**0.024**
	AL	2.7 (2.5–3.0)	2.5 (2.1–3.0)	0.693
	LIR	2.5 (2.1–3.0)	3.5 (2.6–3.5)	0.066
	OI	3.0 (2.6–3.0)	3.5 (2.6–3.5)	0.082
PEP-3	PVC	57.5 (35.2–79.7)	34.5 (24.7–53.5)	0.140
	EL	39.5 (29.2–46.0)	28.5 (20.2–34.5)	0.120
	RL	36.0 (29.0–56.2)	28.5 (20.2–46.5)	0.289
	FMS	59.0 (42.0–76.7)	46.0 (29.7–58.2)	0.273
	GM	56.0 (36.2–66.5)	49.5 (34.2–37.5)	0.623
	VMI	46.5 (34.5–64.5)	47.0 (20.2–59.5)	0.521

Scores are in median (first-third quartile); significant differences are in bold. Legend: CARS-2: RP: Relationship with people; IM: Imitation; ER: Emotional response; UB: Use of the body; UO: Use of objects; AC: Adaptation to changes; VR: Visual response; AR: Auditory response; TS: Taste, smell, and use and response to touch; FA: Fear and apprehension; VC: Verbal communication; NVC: Not verbal communication; AL: Activity level; LIR: Level and consistency of intellectual response; OI: Overall impression. PEP-3 = PVC: pre-verbal cognitive; EL: expressive language; RL: receptive language; FMS: fine motor skills; GM: global motor; VMI: visual-motor imitation.

**Table 4:** ANCOVA results for each covariance model.

Assessment		Group Coefficient				Adjusted R^2^
		Estimate	Std. Error	t Value	*p* Value	
CARS-2	TOTAL	−0.36	0.35	−1.04	0.31	0.97
	RP	0.03	0.07	0.42	0.68	0.90
	IM	<0.01	0.09	0.02	0.98	0.89
	ER	0.02	0.04	0.65	0.52	0.98
	UB	0.02	0.11	0.16	0.87	0.75
	AC	<−0.01	0.08	−0.01	0.99	0.92
	VR	−0.07	0.14	−0.51	0.62	0.49
	AR	−0.21	0.12	−1.74	0.10	0.68
	TS	−0.14	0.06	−2.30	0.03	0.89
	FA	0.11	0.11	0.94	0.36	0.75
	AL	−0.17	0.10	−1.67	0.11	0.67
	LIR	0.03	0.06	0.41	0.68	0.92
	OI	0.03	0.06	0.43	0.67	0.89
PEP-3	PVC	1.73	3.50	0.49	0.63	0.87
	EL	3.94	2.09	1.88	0.08	0.82
	RL	2.04	2.83	0.72	0.48	0.84
	FMS	2.35	3.82	0.62	0.55	0.76
	GM	18.94	6.13	3.09	<0.01	0.85
	VMI	3.26	4.56	0.71	0.48	0.67

Legend: RP: Relationship with people; IM: Imitation; ER: Emotional response; UB: Use of the body; AC: Adaptation to changes; VR: Visual response; AR: Auditory response; TS: Taste, smell, and use and response to touch; FA: Fear and apprehension; AL: Activity level; LIR: Level and consistency of intellectual response; OI: Overall impression. PEP-3 = PVC: pre-verbal cognitive; EL: expressive language; RL: receptive language; FMS: fine motor skills; GM: global motor; VMI: visual-motor imitation.

## Data Availability

The data presented in this study are available on request from the corresponding author.
